# A revision of the purse-web spider genus *Calommata* Lucas, 1837 (Araneae, Atypidae) in the Afrotropical Region

**DOI:** 10.3897/zookeys.95.745

**Published:** 2011-05-04

**Authors:** René Fourie, Charles R. Haddad, Rudy Jocqué

**Affiliations:** 1 Department of Zoology & Entomology, University of the Free State, P.O. Box 339, Bloemfontein 9300, South Africa; 2 Section of Invertebrates, Royal Museum for Central Africa, B-3080 Tervuren, Belgium; 3 Quintiles International, 196 Nelson Mandela Drive, Brandwag, Bloemfontein 9301, South Africa

**Keywords:** conservation, endemic, Mygalomorpha, new species, revalidation

## Abstract

The purse-web spider genus *Calommata* Lucas, 1837 is revised in the Afrotropical Region. Following examination of the female type material, *Calommata transvaalica* Hewitt, 1916 is removed from synonymy with *Calommata simoni* Pocock, 1903 and revalidated. The females of both species are redescribed and their males described for the first time. While *Calommata simoni* is very widespread across tropical Africa, *Calommata transvaalica* is endemic to northern South Africa. Four new species are described, all known only from males: *Calommata megae* **sp. n.** (Zimbabwe), *Calommata meridionalis* **sp. n.** (South Africa), *Calommata namibica* **sp. n.** (Namibia) and *Calommata tibialis* **sp. n.** (Ivory Coast and Togo). Notes are presented on the biology of each species.

## Introduction

The spider family Atypidae includes a small group of distinctive Mygalomorpha, commonly known as purse-web spiders. Presently it comprises three genera, *Atypus* Latreille, 1804, *Calommata* Lucas, 1837 and *Sphodros* Walckenaer, 1835, representing 43 species in Africa, Europe, Asia and North America (Platnick 2010). Morphologically, the family possesses many plesiomorphic features, yet they also have some derived features in both their morphology and technique of prey capture (Gertsch and Platnick 1980). Atypids are burrowing spiders that line their burrows with silk, with an upper section forming a tough web level with the ground, which is expanded and camouflaged as a trap for wandering arthropods (Jocqué and Dippenaar-Schoeman 2006).

Atypidae represents an ancient branch of the Mygalomorpha, with morphological and molecular phylogenetic analyses placing the family basally in the suborder, sister to the Antrodiaetidae in the clade Atypoidina = Atypoidea (Raven 1985, Goloboff 1993, Hedin and Bond 2006, Ayoub et al. 2007). Unfortunately, previous phylogenies have not included African *Calommata*, and thus their relationships to species from other regions and the biogeographical origins of *Calommata* remain totally unexplored.

The Atypidae, with a residual dorsal abdominal scutum in males, can be characterised by (Raven 1985): 1) the extremely elongate, curved inner portion of the maxillary lobes (Figs 10, 11), 2) the broad and obliquely truncated posterior median spinnerets (Figs 32, 35), 3) the rotated orientation of the maxillae (Figs 12–19), and 4) the teeth on the paired and unpaired claws of males and females that are raised on a common process, giving the appearance of a single multipectinate tooth (Raven 1985). However, the latter character is not applicable to the *Calommata* investigated here, neither in males (Figs 23, 27) nor females. Within the family, the three genera can be separated by palpal, spermathecal and sternal morphology, discussed in the diagnosis below.

While the taxonomy of the Atypidae of North America (Gertsch and Platnick 1980), Europe (Kraus and Baur 1974, Schwendinger 1990) and Asia (Schwendinger 1990, Oliger 1998, Tanikawa 2006, Zhu et al. 2006, Levy 2007) has been quite thoroughly studied, the African fauna has not yet been subjected to a modern revision, the last taxonomic paper of note having been published more than 40 years ago (Benoit 1967). Similarly, considerable information is available on the biology (e.g. Clark 1969, Kraus and Baur 1974, Gertsch and Platnick 1980, Coyle and Shear 1981, Anderson 1987, Edwards and Edwards 1990), microhabitat preferences (Řezáč et al. 2007, Řezáč 2009), dispersal (Coyle 1983, Coyle et al. 1985), population genetics (Pedersen and Loeschcke 2001) and karyology (Řezáč et al. 2006) of *Atypus* and *Sphodros*, while considerably less information is available on *Calommata*, particularly the African species, and relates mainly to habitat, burrow structure and prey capture behaviour (Van Dam and Roberts 1917, Blandin 1971, Charpentier 1995, Sato et al. 2007).

Until now, *Calommata* was thought to be represented in the Afrotropical Region by a single species described from Cameroon, *Calommata simoni* Pocock, 1903 (Benoit 1967, Gertsch and Platnick 1980, Dippenaar-Schoeman and Jocqué 1997, Dippenaar-Schoeman 2002, Jocqué and Dippenaar-Schoeman 2006). A second species, *Calommata transvaalicus* Hewitt, 1916, described from Roodeplaat, South Africa, was synonymised with *Calommata simoni* by Benoit (1967). The remaining six species in the genus, *Calommata fulvipes* (Lucas, 1835), *Calommata obesa* Simon, 1886, *Calommata pichoni* Schenkel, 1963, *Calommata signata* Karsch, 1879, *Calommata sundaica* (Doleschall, 1859) and *Calommata truculenta* (Thorell, 1887), were all described from East Asia, although *Calommata sundaica* has recently been recorded from Israel (Levy 2007).

In this paper the *Calommata* of the Afrotropical Region are revised, *Calommata transvaalica* is removed from synonymy with *Calommata simoni*, and four new species are described. While the biology of *Calommata simoni* and *Calommata transvaalica* has been studied (Van Dam and Roberts 1917, Blandin 1971, Charpentier 1995), little is known about the habits of the new species, which is largely due to the general scarcity of these spiders. We suspect that the biological studies related to *Calommata simoni* do, in fact, refer to *Calommata tibialis* sp. n., based on available material from one study and contrasting habitat preferences of the two species. By resolving the taxonomy of the Afrotropical fauna, the representative species can be included in future phylogenetic analyses and conservation assessments.

## Material and methods

All spiders were studied under an Olympus SZX10 stereomicroscope under 10× magnification. Measurements (body and legs) were taken with a measuring eyepiece and are given in millimetres (mm). The proportional indices used follow Levy (2007) and are the carapace index (length divided by width), and patella-tibia index (combined length of the patella and tibia segments of the first leg divided by the length of the carapace). Total length is given as the length from the front of the chelicerae to the tip of the abdomen, excluding the spinnerets. A range of total length measurements is provided for each species from the material examined. Where geographical locality data was not provided on specimen labels or was not available in collection databases, geographical co-ordinates were searched for in the Global Gazetteer Version 2.2 (http://www.fallingrain.com) or using Google Earth Version 5.0 (http://www.google.com/earth), and are indicated in square parenthesis.

Material of male specimens of *Calommata meridionalis* sp. n., *Calommata namibica* sp. n., *Calommata simoni* and *Calommata tibialis* sp. n. was prepared for Scanning Electron Microscopy (SEM). Specimens were transferred to absolute (100 %) ethanol and left overnight. After drying in hexamethyldisilazane (HMDS), the specimens were glued to rounded aluminium rivets using two-sided copper strips and then coated with gold for examination using a JEOL JSM-6480 scanning electron microscope at the MRAC. Digitised micrographs were taken.

Digital photographs of the dorsal habitus of a *Calommata simoni* female were taken with a Leica MZ16 and stacked using the LAS automontage software at the MRAC. Digital photographs of the dorsal habitus of the remaining species and a *Calommata simoni* male were taken using a Nikon Coolpix 8400 mounted on a Nikon SMZ800 stereomicroscope at the UFS. The extended focal range images were stacked using CombineZM software to increase depth of field (http://www.hadleyweb.pwp.blueyonder.co.uk/).

### Abbreviations:

AL abdomen length

ALS anterior lateral spinneret(s)

AMNH American Museum of Natural History (New York, U.S.A.)

AW abdomen width

BMNH BritishNatural History Museum (London, U.K.)

CL carapace length

CW carapace width

MRAC Royal Museum for Central Africa (Tervuren, Belgium)

NCA National Collection of Arachnida, Plant Protection Research Institute (Pretoria, South Africa)

NMBA National Museum (Bloemfontein, South Africa)

NMSA Natal Museum (Pietermaritzburg, South Africa)

PLS posterior lateral spinneret(s)

PMS posterior median spinneret(s)

UFS University of the Free State (Bloemfontein, South Africa)

TL total length

TMSA Ditsong National Museum of Natural History (Pretoria, South Africa)

USNM National Museum of Natural History (Washington D.C., U.S.A.)

## Taxonomy

**Family Atypidae**

**Subfamily Atypinae**

### Genus Calommata

Lucas, 1837

http://species-id.net/wiki/Calommata

#### Type species.

*Pachyloscelis fulvipes* Lucas, 1835 from Java and Sumatra

#### Diagnosis.

**Figures 1–8.**
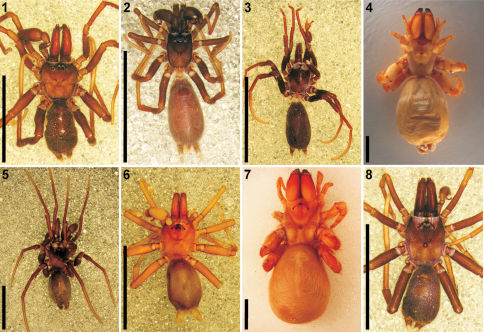
Dorsal habitus of Afrotropical *Calommata* species **1** *Calommata megae* sp. n., male (Harare, Zimbabwe) **2** *Calommata meridionalis* sp. n., male (Erfenis Dam, South Africa) **3** *Calommata namibica* sp. n., male (Etosha, Namibia) **4** *Calommata simoni* Pocock, female (Galim, Cameroon) **5** same, male (Dja Reserve, Cameroon) **6** *Calommata tibialis* sp. n., male (Bassari–Sokodé, Togo) **7** *Calommata transvaalica* Hewitt, female (Blouberg, South Africa) **8** same, male (Groenkloof, South Africa). Scale bars: 5mm.

**Figures 9–19.**
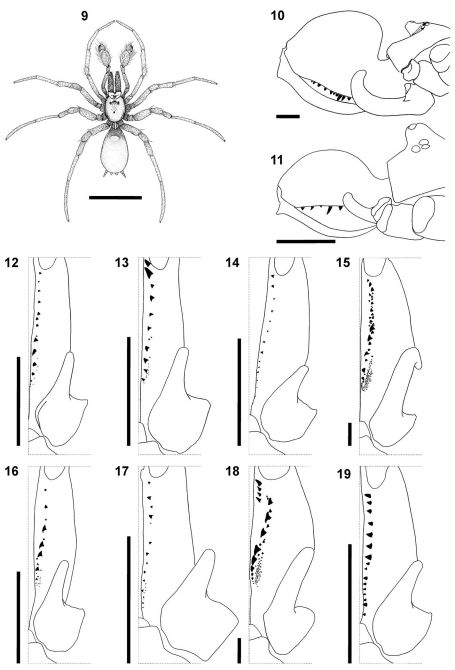
Somatic morphology of *Calommata tibialis* sp. n., male **9, 17** *Calommata simoni* Pocock, female **10, 15 **and male **11, 16** *Calommata megae* sp. n., male **12** *Calommata meridionalis* sp. n., male **13** *Calommata namibica* sp. n., male **14**and *Calommata transvaalica* Hewitt, female **18** and male **19: 9** dorsal habitus **10, 11** lateral view of chelicera, endites and anterior of carapace **12–19** left chelicera, ventral view, indicating dentition. Scale bars: 5mm (9), 1mm (10–19).

*Calommata* can be distinguished from *Atypus* and *Sphodros* by three main characteristics: 1) Spermathecal structure. In *Atypus*, there are two broad plates each bearing two or more small receptacula (Schwendinger 1990: figs 14–25), in *Sphodros* the four spermathecae are each highly coiled and without distinct receptacula (e.g. Gertsch and Platnick 1980: fig. 29), whereas in *Calommata* there are four spermathecae (Fig. 62), each bearing several closely packed terminal receptacula positioned in a cauliflower-like arrangement,). However, the latter definition was based only on the spermathecal structure of three of the seven *Calommata* species (Gertsch and Platnick 1980), and variation does indeed occur within the spermathecal structure of the genus. The female genitalia of *Calommata transvaalica* only bear one pair of oval spermathecae (Fig. 69). 2) Male palpal cymbium structure. In *Calommata* the palpal cymbium is short and truncate (Fig. 48), while in *Atypus* and *Sphodros* it is short and acuminate (Gertsch and Platnick 1980: figs 15, 25; Raven 1985). 3) Labiosternal suture. In *Calommata* the labiosternal suture is positioned anteriorly on the sternum (Levy 2007: fig. 8), but is considered by Gertsch and Platnick (1980) to be absent and by Raven (1985) to have migrated posteriorly in *Atypus* and *Sphodros* (see Gertsch and Platnick 1980: figs 14, 22). Further morphological characters unique to *Calommata* (Gertsch and Platnick 1980) include the bipartite, longitudinal thoracic groove (fovea) (Figs 1–9); basal ledge on the outer surface of the fangs of both sexes, the posteriorly positioned ocular tubercle, enormously elongated endites and dorsally expanded chelicerae (Figs 10, 11); short leg I, particularly the femur, and flattened palpal tibia and tarsus of females (Figs 4, 7); and the greatly elongate tarsi of legs III and IV of males, which are clearly pseudosegmented (e.g. Figs 3, 5, 30).

#### Description.

**Figures 20–31.**
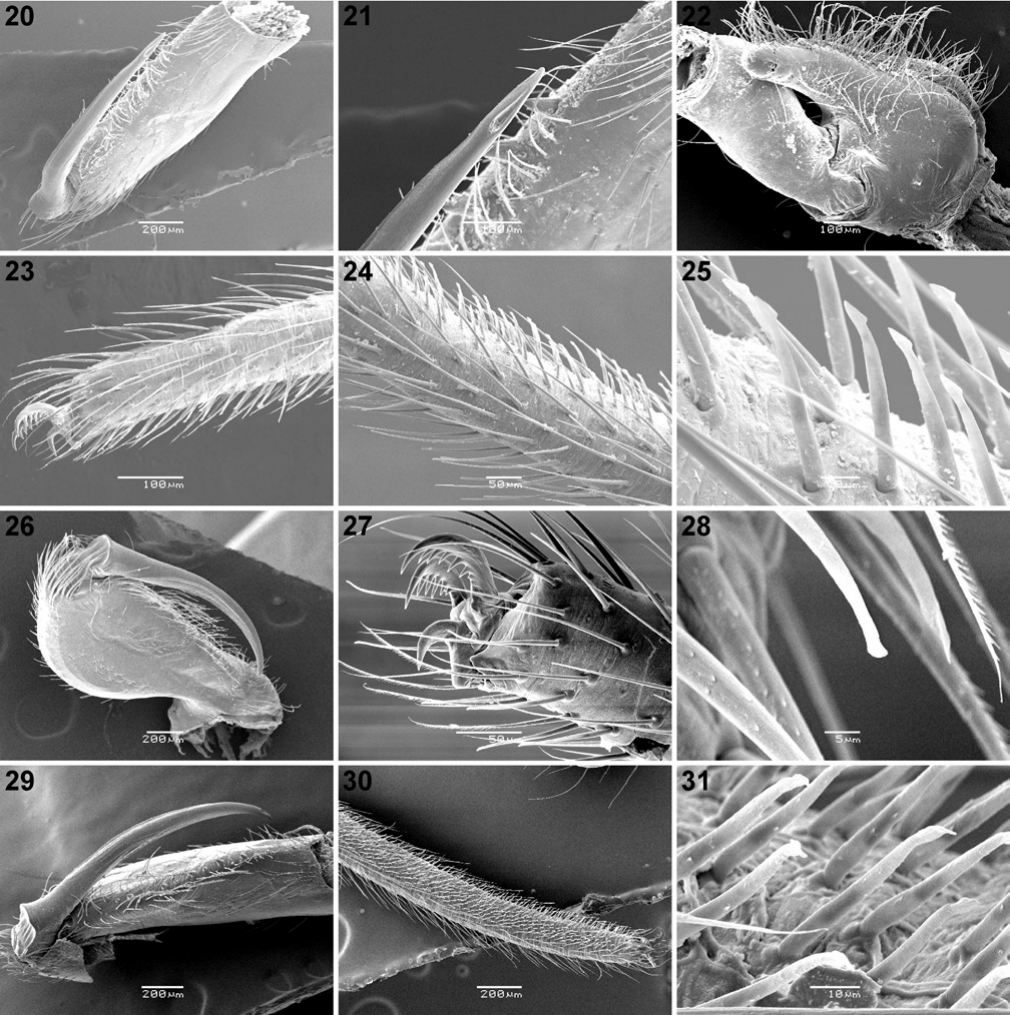
Scanning electron micrographs of *Calommata meridionalis* sp. n. **20–25** *Calommata simoni* Pocock **26–28** and *Calommata tibialis* sp. n. **29–31** males **20, 29** chelicera in ventral view **21** tip of fang **22** right endite, ventral view **23, 27** tarsus and claw, leg I (note pseudosegmentation of tarsus) **24, 30** tarsus IV, lateral and ventral view (note pseudosegmentation of tarsus) **25, 28, 31** detail of ventral scopulate setae on tarsus IV **26** chelicera in prolateral view.

**Figures 32–40.**
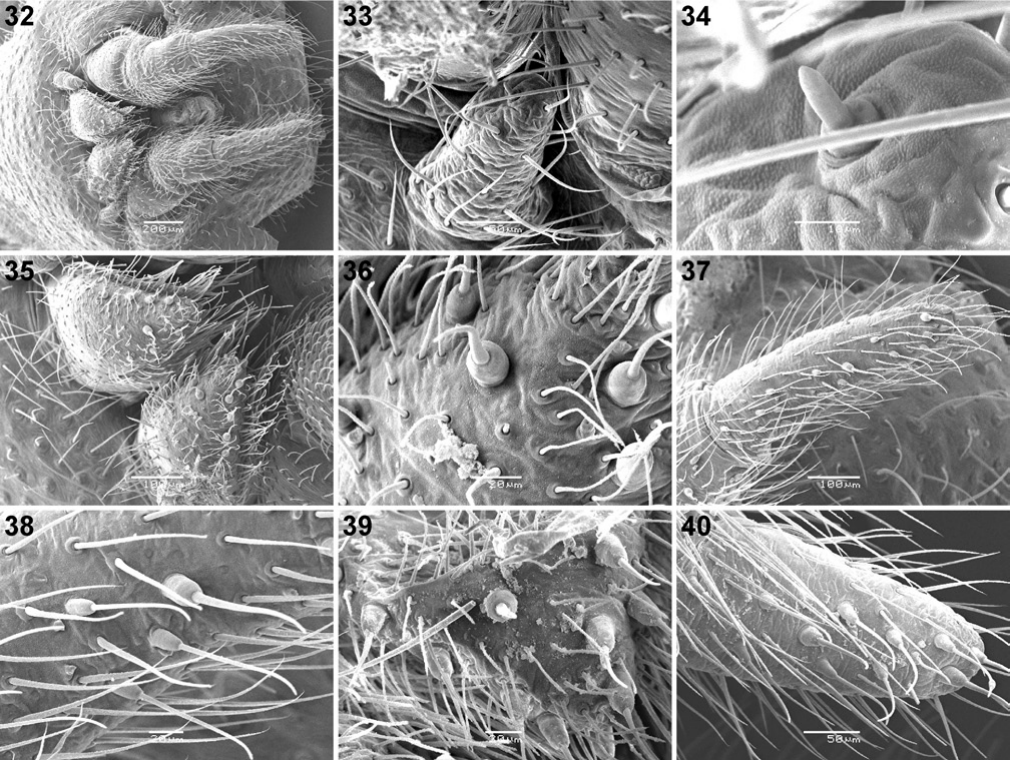
Scanning electron micrographs of *Calommata simoni* Pocock **32–38** and *Calommata meridionalis* sp. n. **39, 40** male spinnerets **32** spinneret field, ventral view **33** ALS **34** detail of single ALS spigot **35** PMS **36, 39** detail of PMS spigots **37, 40** PLS **38** detail of PLS spigots.

**Figures 41–52.**
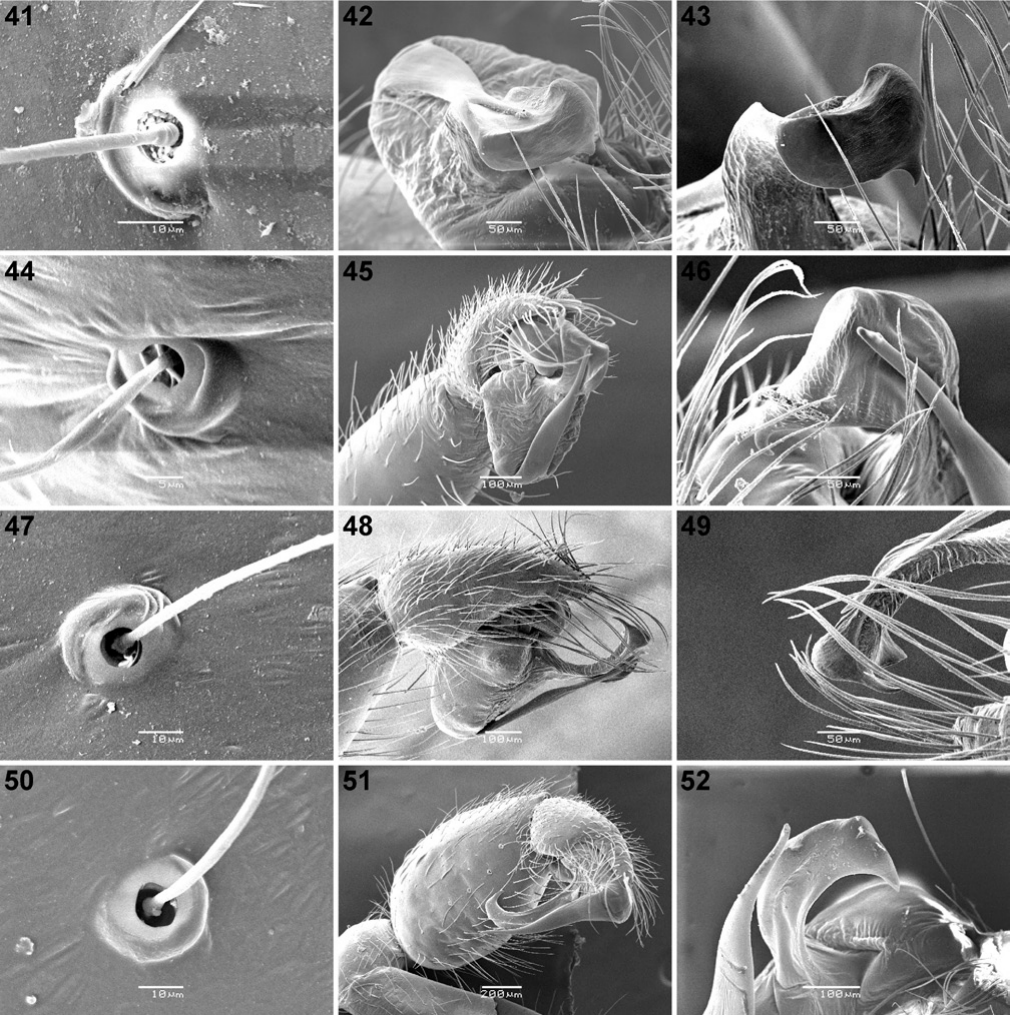
Scanning electron micrographs of *Calommata meridionalis* sp. n. **41–43**, *Calommata namibica* **44–46**, *Calommata simoni* Pocock **47–49** and *Calommata tibialis* sp. n. **50–52** males **41, 44, 47, 50** trichobothrial bases on palpal tarsus **42, 43, 46, 49, 52** distal end of conductor and embolus **45** palpal organ in prolateral ventral view **48, 51** palpal organ in prolateral view.

Medium to large sexually dimorphic spiders (Figs 1–9), females 18.60–33.40 in length and males 5.10–9.35 in length. Carapace with an anterior, strongly elevated median ocular tubercle (Figs 10, 11), with a flattened posterior part traversed by a longitudinal thoracic furrow with a small deep pit in the middle (Figs 1–8). Three faint lines run from the ocular tubercle, converging at the fovea. Chelicerae massive, dorsally expanded with flattened sides, bearing sharp teeth usually in one (rarely two) rows, and a long arched fang with a slit-like distal opening and distinctive basal ledge along its outer margin (Figs 10–19, 20, 21, 26, 29). Cheliceral dentition of both sexes often variable between specimens and even between opposing chelicerae of a specimen. The general dentition pattern of each species and sex is indicated in Figs 12–19. Chelicerae of females have larger, more numerous teeth and an extensive field of tiny denticles retrolateral of the teeth row near the cheliceral base (Figs 15, 18). Chilum single, large, varying in shape, oval, rectangular or pentagonal (Figs 1–8). Endites strongly elongated and curved upwards on the prolateral side (Figs 10, 11, 22). Sternum with a distinctive labio-sternal suture anteriorly. Legs of females short and stout, leg formula of females 4231. Legs of males of normal size, with the tarsi pseudosegmented (Figs 23, 24, 30) and tarsi of legs III and IV distinctly longer (Figs 1–3, 5, 6, 8, 9); leg formula of males usually 4321, rarely 4132. Tarsi with three claws, paired claws of males with single row of teeth and unpaired claw without teeth (Figs 23, 27), *contra* Raven (1985: 123). Tarsi ventrally scopulate, consisting of pointed setae (Figs 25, 31) or setae with a rounded tip (Fig. 28). Abdomen of male with small, distinctive anterior dorsal scutum, absent in females (Figs 1–8). Venter with three pairs of spinnerets, examined in detail in males (Figs 32–40) but not females; ALS small and finger-like, single-segmented, with a single short, stout distal fused spigot (Figs 32–34); PMS small and subtriangular, single-segmented, with several fused and articulated spigots in distal half of segment (Figs 32, 35, 36, 39); PLS large, elongate, three segmented, distal segment digitiform, with several fused and articulated spigots in distal half of second and entire ventral surface of third segment (Figs 32, 37, 38, 40). Anal tubercle widely separated from the spinnerets. Female palp short, with flattened tibia and tarsus (Fig. 7). Female epigyne forming broad, weakly sclerotised plate in ventral view; epigyne with two pairs of spermathecae, median pair subrectangular and rounded anteriorly, and lateral pair subtriangular (Fig. 62), or with single pair of transversely oval spermathecae (Fig. 69). Male palp with swollen tibia, bearing a retrolateral ventral row of several trichobothria, the bases of which are raised to one side; palpal tegulum with curved broad conductor with tooth on its dorsal surface, embolus straight, tapering towards tip (Figs 41–52).

##### Key to the Afrotropical Calommata

[females of *Calommata megae* sp. n., *Calommata meridionalis* sp. n., *Calommata namibica* sp. n. and *Calommata tibialis* sp. n. unknown]

**Table:** 

1	Females	2
–	Males	3
2	Chelicerae with a single row of teeth along promargin running from fang base close to cheliceral base (Fig. 15); epigyne with two pairs of spermathecae (Fig. 62) (tropical Africa)	*Calommata simoni*
–	Chelicerae with an additional row of 2–4 teeth along promargin placed close to fang base, prolateral of main teeth row (Fig. 18); epigyne with one pair of transversely oval spermathecae (Fig. 69) (South Africa)	*Calommata transvaalica*
3	CL subequal to CW	4
–	Carapace clearly longer than wide, CL>1.15CW	5
4	Embolus and conductor in ventral view directed obliquely and retrolaterally towards endites (Fig. 54); palpal tibia clearly longer than wide (Fig. 53), patella-tibia index ~1.3 (Zimbabwe)	*Calommata megae*
–	Embolus and conductor in ventral view directed obliquely and retrolaterally towards distal end of chelicerae (Fig. 67); palpal tibia swollen, only slightly longer than wide (Fig. 66), patella-tibia index ~0.9 (Cote d’Ivoire and Togo)	*Calommata tibialis*
5	Embolus and conductor in ventral view directed transversely across palpal axis	6
–	Embolus and conductor in ventral view directed obliquely relative to palpal axis	7
6	Chelicerae with one or two very large teeth near fang base, remaining teeth distinctly smaller; several small denticles retrolateral of teeth row near base of chelicerae (Fig. 13); cymbium tip in ventral view broad and rounded (Fig. 57) (South Africa)	*Calommata meridionalis*
–	Chelicerae without one or two large teeth near fang base, teeth gradually decreasing in size from fang base to cheliceral base; denticles absent near base of chelicerae (Fig. 19); cymbium tip in ventral view tapering to rounded point (Fig. 71) (South Africa)	*Calommata transvaalica*
7	Cheliceral teeth variable in size, with at least three large teeth in midsection of teeth row and several denticles retrolateral of teeth row (Fig. 16); conductor and embolus not projecting beyond cymbial margin in ventral view (Fig. 64); conductor with distinct tooth and second ridge resembling a tooth distally on its dorsal surface (Fig. 49) (tropical Africa)	*Calommata simoni*
–	Cheliceral teeth tiny, subequal, without denticles retrolateral of teeth row (Fig. 14); conductor and embolus projecting far beyond cymbial margin in ventral view (Fig. 60); conductor with single tooth distally on its dorsal surface (Fig. 46) (Namibia)	*Calommata namibica*

### Calommata megae

Fourie, Haddad & Jocqué sp. n.

urn:lsid:zoobank.org:act:ED324676-F4FB-4F0D-816E-4670E7EE2577

http://species-id.net/wiki/Calommata_megae

Figs 1 12 53–55

#### Type material. Holotype male.

ZIMBABWE: Harare, Highlands [17°48'S, 31°05'E], 13.I.2003, on soil, by hand, M. Cumming (NCA 2004/362).

#### Other material examined.

ZIMBABWE: 1imm.: same locality as holotype, 21.II.2000, in garden, M. Cumming (NCA 2004/1361).

#### Diagnosis.

**Figures 53–55.**
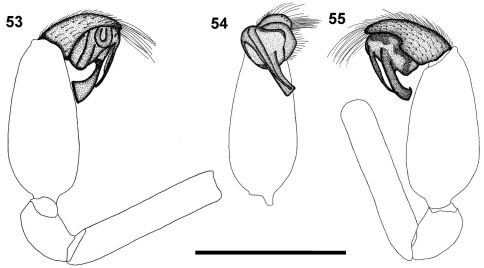
Left palp of male *Calommata megae* sp. n. **53** prolateral view **54** ventral view **55** retrolateral view. Scale bars: 1mm.

The male of this species is recognised by the conductor that narrows and makes a half twist before broadening distally, and the obliquely orientated embolus and conductor (Figs 53–55). This species shares with *Calommata tibialis* sp. n. the carapace that is subequal in length and width (longer than wide in the other four species).

#### Etymology.

The specific epithet is a patronym in honour of the collector of the holotype, Meg Cumming, in recognition of her contributions to African arachnology, particularly in Zimbabwe.

#### Description.

**Male holotype.** Measurements: CL 2.00, CW 2.05, AL 2.95, AW 1.80, TL 7.00. Length of leg segments, and total: I 2.40 + 0.90 + 1.70 + 1.90 + 1.70 = 8.60; II 2.15 + 0.95 + 1.40 + 2.20 + 2.15 = 8.85; III 1.80 + 0.95 + 1.00 + 2.15 + 3.55 = 9.45; 2.35 + 1.10 + 1.28 + 2.50 + 4.35 = 11.58. Carapace index 0.98; patella-tibia index 1.30.

Carapace and chelicerae brown in colour (Fig. 1). Carapace flat and robust. Median ocular tubercle raised, darker in colour.Chelicerae with a single row of small teeth, increasing in size from fang base to base of chelicerae, with a few denticles near base of chelicerae (Fig. 12). Sternum and coxae light brown, remainder of legs dark brown, fading to light yellow-brown at tarsi. Legs weakly covered with bristles; prolateral side of patellae, tibiae and metatarsi of legs II–IV covered with spinules (thicker and shorter than bristles). Abdomen dark brown, with an irregular brown scutum present anteriorly (Fig. 1). Palpal cymbium short with rounded distal margin; embolus and conductor orientated obliquely, pointing retrolaterally towards base of chelicerae; conductor narrow, making a half twist before broadening distally, with single small curved tooth on its dorsal surface distally; embolus short and straight, slightly curved near tip (Figs 53–55).

**Female.** Unknown.

#### Distribution.

Known only from the type locality (Fig. 73).

#### Biology.

Poorly known. The holotype male was collected in mid-summer on the soil surface. The second instar juvenile specimen was captured while ballooning and landing on the porch of a house (M. Cumming, pers. comm.). Amongst atypids, ballooning has previously been recorded in both *Atypus* and *Sphodros* (Wiehle 1953 cited in Pedersen and Loeschcke 2001, Coyle 1983, Coyle et al. 1985).

### Calommata meridionalis

Fourie, Haddad & Jocqué sp. n.

urn:lsid:zoobank.org:act:7E2F6F05-6995-4C3B-AC59-30A0CE451914

http://species-id.net/wiki/Calommata_meridionalis

Figs 2 13 20–25 39 43 56–58

#### Type material.

**Holotype male.** SOUTH AFRICA: *Free State Province*: Erfenis Dam Nature Reserve, 28°29.888'S, 26°48.488'E, 21.IX–22.X.2005, pitfalls, unburned site 1, C. Haddad, S. Otto & R. Poller (NCA 2009/3488).

#### Paratypes.

SOUTH AFRICA: *Free State Province*: 3♂: Bloemfontein, National Botanical Gardens, 29°03.006'S, 26°12.701'E, 27.X–16.XI.2009, pitfalls, grassland, C. Haddad (NMBA 13981); 7♂: Same data, 16–21.XI.2009 (NMSA 22616); 1♂: Same locality, 21.XI–7.XII.2009, pitfalls, grassland, C. Haddad & R. Fourie (MRAC 229029); 1♂: Erfenis Dam Nature Reserve, 28°30.373'S, 26°48.437'E, 21.IX–22.X.2005, pitfalls, burned site 1, C. Haddad, S. Otto & R. Poller (NCA 2009/3663); 4♂: Same locality, 28°30.134'S, 26°48.427'E, 22.X–22.XI.2005, pitfalls, burned site 2, C. Haddad, S. Otto & R. Poller (NCA 2007/3142); 1♂: Same locality, 28°29.741'S, 26°48.065'E, 21.IX–22.X.2005, pitfalls, unburned site 3, C. Haddad, S. Otto & R. Poller (NCA 2009/3664); 1♂: Oranjeville district, Vaal Dam, 26°59.523'S, 28°15.737'E, 1–29.X.2009, pitfalls, grassland, R. Fourie & A. Grobler (NCA 2009/3539).

#### Other material examined.

SOUTH AFRICA: *Free State Province*: 1♂: Erfenis Dam Nature Reserve, 28°30.134'S, 26°48.427'E, 21.IX–22.X.2005, pitfalls, burned site 2, C. Haddad, S. Otto & R. Poller (MRAC, prepared for SEM).

#### Diagnosis.

The male of this species can be easily recognised from African congeners by the presence of one or two very large teeth at the fang base (Fig. 13), and the transversely orientated curved embolus with distally broadened conductor bearing a single tooth on its dorsal surface (Figs 42, 43, 57). The raised median ocular tubercle is broader than in the other species.

**Figures 56–58.**
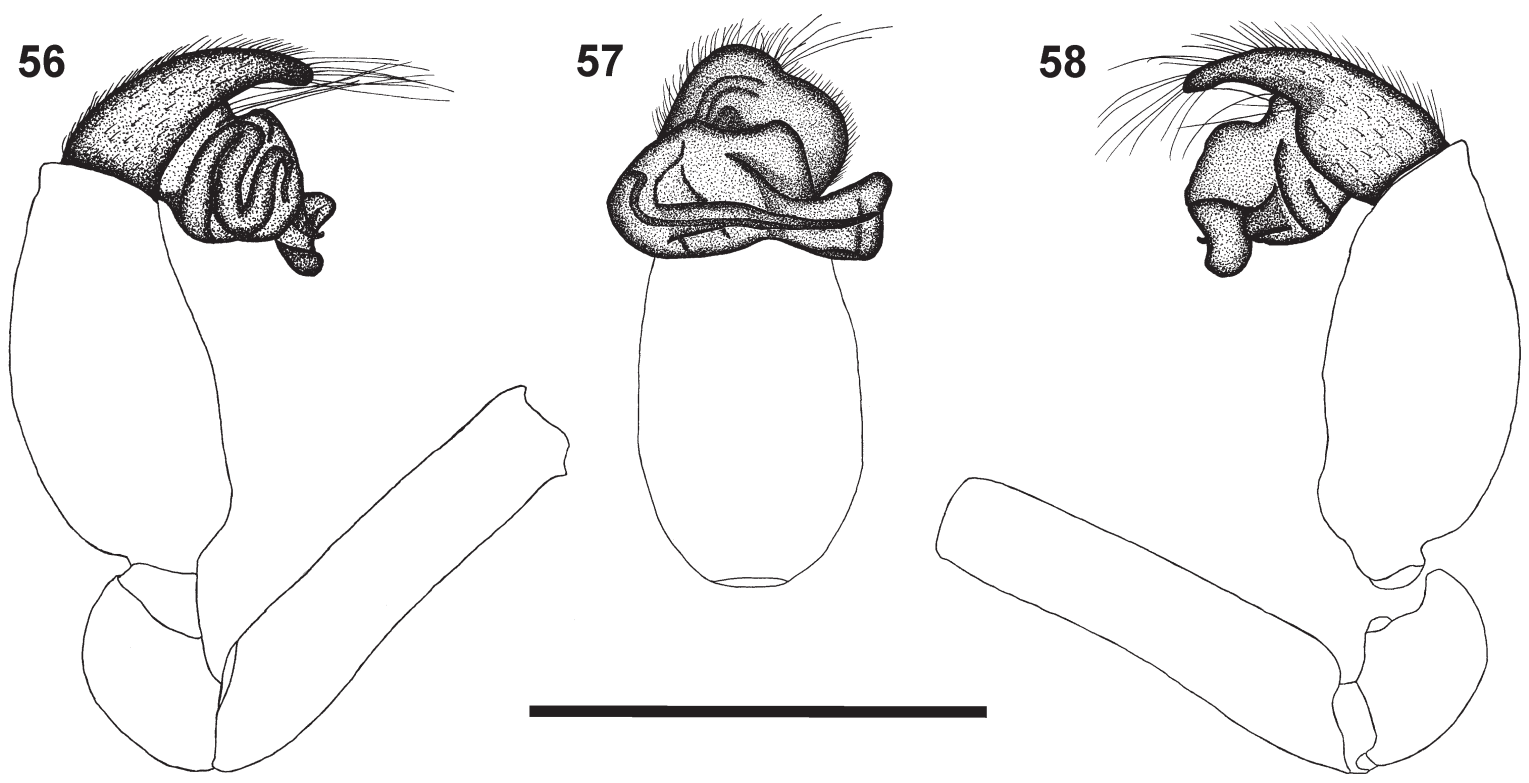
Left palp of male *Calommata meridionalis* sp. n. **56** prolateral view **57** ventral view **58** retrolateral view. Scale bars: 1mm.

#### Etymology.

This specific epithet is Latin for southern, referring to the distribution of the species, southernmost in the genus.

#### Description.

**Male holotype.** Measurements: CL 1.60, CW 1.25, AL 3.15, AW 1.68, TL 6.35 (5.60–7.80). Length of leg segments, and total: I 1.40 + 0.55 + 1.00 + 1.15 + 1.25 = 5.35; II 1.30 + 0.55 + 0.85 + 1.24 + 1.45 = 5.39; III 1.05 + 0.64 + 0.64 + 1.20 + 2.40 = 5.93; IV 1.45 + 0.64 + 0.75 + 1.44 + 2.90 = 7.18. Carapace index 1.28; patella-tibia index 0.97.

Carapace and chelicerae dark brown in colour (Fig. 2). Median ocular tubercle raised, darker in colour; median ocular tubercle broader than in other species. Chelicerae with single prolateral row of teeth, one very large tooth close to fang base, sometimes accompanied by second large tooth, remaining teeth distinctly smaller and subequal in size, with several denticles retrolateral of teeth row close to cheliceral base (Fig. 13). Sternum and coxae pale brown, remainder of legs brown, gradually fading to yellow at tarsi. Legs weakly covered with bristles; prolateral side of patellae, tibiae and metatarsi of legs II–IV covered with spinules. Abdomen grey-brown, with a round brown scutum anteriorly (Fig. 2). Palpal cymbium short with rounded distal margin; embolus and conductor orientated transversely across palpal axis, pointing retrolaterally; conductor broadened distally, with a single small tooth on its dorsal surface distally; embolus long, slightly curved in a S-form along its length (Figs 42, 43, 56–58).

**Female.** Unknown.

#### Distribution.

Endemic to central and northern Free State Province, South Africa (Fig. 73).

#### Biology.

The species was collected exclusively by pitfalls in spring and early summer (September to early December) in the Grassland Biome of South Africa. Specimens were only collected in dark vertic clay and loamy-clay soils and not from sites with sandy soils. Most of the specimens were collected from sites near to freshwater streams and dams. Despite exhaustive attempts to locate burrows in the vicinity of pitfall sites (Erfenis Dam Nature Reserve and Botanical Gardens) none could be found.

### Calommata namibica

Fourie, Haddad & Jocqué sp. n.

urn:lsid:zoobank.org:act:099D7F5A-BBF3-482E-B570-3D8053C88E93

http://species-id.net/wiki/Calommata_namibica

Figs 3 14 44–46 59–61

#### Type material. Holotype male and paratype male.

NAMIBIA: Etosha National Park, Beisebvlakte, 18°32'S, 17°02'E, 10–14.XI.1996, A. Russell-Smith (MRAC 215409).

#### Other material examined.

None.

**Diagnosis.** The male of this species can be recognised by the tiny cheliceral teeth in a single row, without retrolateral denticles (Fig. 14), and the obliquely orientated conductor and embolus projecting far beyond the retrolateral cymbial margin (Fig. 60).

#### Etymology.

The specific epithet refers to the country of the type locality.

#### Description.

**Male holotype.** Measurements: CL 1.80, CW 1.24, AL 2.75, AW 1.54, TL 6.30 (5.10–6.30). Length of leg segments, and total: I 1.21 + 0.50 + 0.83 + 1.09 + 1.35 = 4.98; II 1.40 + 0.51 + 0.62 + 1.13 + 1.40 = 5.06; III 1.05 + 0.49 + 0.47 + 1.16 + 1.95 = 5.12; IV 1.44 + 0.60 + 0.65 + 1.38 + 2.40 = 6.47. Carapace index 1.45; patella-tibia index 0.74.

**Figures 59–61.**
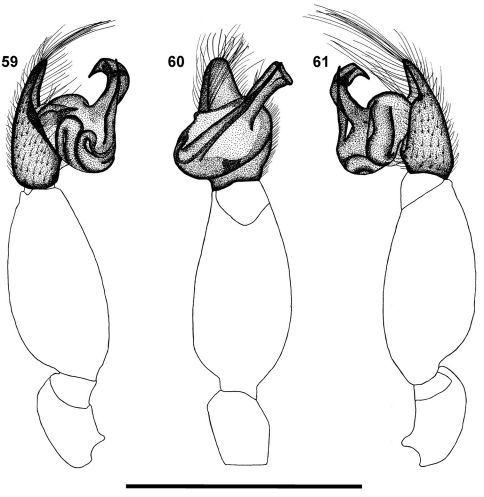
Left palp of male *Calommata namibica* sp. n. **59** prolateral view **60** ventral view **61** retrolateral view. Scale bars: 1mm.

Carapace and chelicerae dark brown (Fig. 3). Median ocular tubercle raised, narrow, darker in colour. Chelicerae with single prolateral row of tiny teeth, without denticles near cheliceral base (Fig. 14). Sternum and coxae light brown, femora dark brown; subsequent segments fading to light yellow at tarsi. Legs weakly covered with bristles; prolateral side of patellae, tibiae and metatarsi of legs II–IV covered with spinules. Abdomen dark grey, nearly black, with brown scutum present in the anterior half (Fig. 3). Palp with elongate cymbium, with tapering pointed distal margin; embolus and conductor orientated obliquely, pointing retrolaterally and distally, projecting far beyond retrolateral cymbial margin; conductor short, slightly broadened distally, with a very prominent, very long and slender tooth distally on its dorsal surface; embolus long, with slight bend in distal half (Figs 45, 46, 59–61).

**Female.** Unknown.

#### Distribution.

Known only from the type locality (Fig. 73).

#### Biology.

Poorly known. This species was collected in late spring in arid savanna.

### Calommata simoni

Pocock, 1903

http://species-id.net/wiki/Calommata_simoni

Figs 4, 5 10, 11, 15, 16 26–28 32–38 47–49 62–65

Calommata simoni Pocock, 1903: 259; Benoit, 1967: 283, figs 1–4; Kraus 1978: 245, fig. 13; Dippenaar-Schoeman and Jocqué 1997: 52, figs 40a–h; Dippenaar-Schoeman 2002: 23, figs 9, 10a–g ; Jocqué and Dippenaar-Schoeman 2006: 82, figs 18a–h.

#### Type material. Lectotype female.

CAMEROON: Efulen [02°46'N, 10°43'E], G.L. Bates (BMHN, examined).

#### Other material examined.

CAMEROON: 1♀: Galim [07°06'N, 12°28'E], 15.VIII–19.VIII.1971, F. Puylaert (MRAC 143671); 3♂: N of Dja Reserve, 03°41'N, 13°14'E, 8.III.2005, pitfalls, old secondary forest, I. Deblauwe (MRAC 220674); 1♂: Same locality, 8.III.2005, pitfalls, riverine forest, I. Deblauwe (MRAC 220663); 1♂: Same data, 6.V.2005 (MRAC 219754); 6♂: Same locality, 8.III.2005, pitfalls, young secondary forest, I. Deblauwe (MRAC 220659). CONGO D.R.: 1♂: Kisangani, Masako Forest, 00°35'N, 25°11'E, 25.II.2003, J.-L. Juakaly (MRAC 216031); 1♂: Same locality, 11.III.2003, pitfalls, primary forest, J.-L. Juakaly (MRAC 214347); 1♂: Same data, 11.III.2003 (MRAC 214354); 1♂: Same data, 11.III.2003 (MRAC 214385); 1♀: Kisantu, 05°08'S, 15°06'E, 1927, R. Vanderyst (MRAC 5201); 1♀: Zambi [05°51'S, 12°52'E], 1909–1915, American Museum Congo Expedition (AMNH). CÔTE D’IVOIRE: 1♂: Appouesso, Bossematié Forest, 06°35'N, 03°28'W, 19.IX.1994, pitfalls, rain forest, R. Jocqué & N. Séabé (MRAC 202481); 1♂: Same locality, 12.III.1995, pitfalls, forest, R. Jocqué & Tanoh (MRAC 205382); 1♂: Same data, 26.III.1995 (MRAC 205383); 1♂: Mankono, Ranch de la Marahoué, 08°27'N, 06°52'W, III.1980,  riverine forest, J. Everts (MRAC 172117); 1♂: Same data (MRAC 172118); 1♂: Same data (MRAC 172119); 2♂: Same data (MRAC 172120). GUINÉE: 3♂: F.C. de Ziama, 08°24'N, 09°17'W, 22.IV.1998, pitfalls, rain forest, D. Flomo (MRAC 216239); 2♂: Same data, 5.V.1998 (MRAC 216248); 2♂: Same data, 18.V.1998 (MRAC 216249); 1♂: Same data, 14.VI.1998 (MRAC 216247); 1♂: Same data, 14.VI.1998 (MRAC 216250); 1♂: Same data, 14.VI.1998 (MRAC 216251); 1♂: Same data, 31.III.1999 (MRAC 216245); 2♂: Same data, 26.IV.1999 (MRAC 216243); 1♂: Same data, 26.IV.1999 (MRAC 216244); 1♂: Same data, 9.V.1999 (MRAC 216242); 2♂: Same data, 22.V.1999 (MRAC 216240); 1♂: Same data, 17.VI.1999 (MRAC 216241); 1♂: Same data, 31.III.2000 (MRAC 216246). KENYA: 1♂: Kakamega Forest, 00°13'N, 34°54'E, 24.I.2002, pitfalls, D. Shilabira Smith (MRAC 228141); 1♂: Same data, 13.IV.2002 (MRAC 220536). LIBERIA: 1imm.: Mount Coffee, Bensonville [06°29'N, 10°38'W], II.1894, constructs a tube-like nest under a log, collector unknown (USNM). MALAWI: 1♂: Chisasira Forest, 25km S of Chintheche, 11°50'S, 33°13'E, 1.XII.1977, pitfalls, *Brachystegia* woodland, R. Jocqué (MRAC 169498); 1♂: Same data, 1.XII.1977 (MRAC 169499). TANZANIA: 1imm.: Bunduki, Uluguru Mountains, 07°02'S, 37°38'E, 2.V.1957, nest, forest ground in litter, P. Basilewsky & N. Leleup (MRAC 111792).

#### Diagnosis.

**Figures 62–65.**
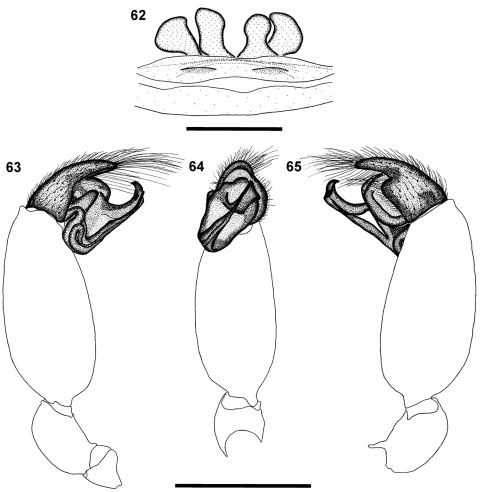
*Calommata simoni* Pocock female genitalia **62** and left palp of male **63–65:** **62** dorsal view **63** prolateral view **64** ventral view **65** retrolateral view. Scale bars: 1mm.

The female of this species has the cheliceral teeth in a single row (Fig. 15), while an additional row is found in *Calommata transvaalica* (Fig. 18). The female genitalia have two pairs of small spermathecae (Fig. 62), while *Calommata transvaalica* only has a single pair of large transverse spermathecae (Fig. 69). The male of this species is recognised by the conductor ending broadly with a prominent tooth and sharp edge, appearing to be a second tooth, on its dorsal surface (Fig. 49). In *Calommata simoni* females the patella-tibia index is double that of *Calommata transvaalica*.

#### Redescription.

**Female** (measurements provided for female lectotype from Efulen, colouration for female from Galim). Measurements: CL 7.70, CW 6.10, AL 13.10, AW 8.85, TL 27.80 (25.80–33.40). Length of leg segments, and total: I 3.95 + 1.62 + 1.75 + 2.10 + 1.38 = 10.80; 3.70 + 2.25 + 1.70 + 2.23 + 1.60 = 11.48; III 3.65 + 2.75 + 1.55 + 1.78 + 1.39 = 11.12; IV 3.90 + 2.90 + 2.10 + 2.15 + 1.50 = 12.55. Carapace index 1.26; patella-tibia index 0.44.

Robustly built with short legs, carapace faded to creamy brown (Fig. 4). Median ocular tubercle raised, narrow, sloping sharply at fovea (Fig. 10). Single median line running from anterior of eye area to approximately middle of chilum. Chelicerae pale orange brown, darker laterally; chelicerae with a single row of small and medium sized teeth along promargin running from fang base close to cheliceral base, with extensive denticle field retrolateral of teeth row near cheliceral base (Fig. 15). Endites strongly elongated prolaterally, strongly curved upwards (Fig. 10). Sternum and legs light yellowish brown. Legs short and stout, leg formula 4231; legs III and IV more robust than legs I and II; leg I without bristles or spinules; leg II with few spinules distally on tibiae, and dorsal and lateral spinules on metatarsi and tarsi; legs III and IV with spinules from patellae to tarsi (mainly dorsal and prolateral) and covered in bristles. Abdomen globose and pale grey, with indistinct median heart marking in anterior half (Fig. 4). Epigyne forming a broad, weakly sclerotised plate ventrally, in dorsal view with two pairs of spermathecae; median pair subrectangular, rounded anteriorly, lateral pair subtriangular (Fig. 62). Female palp short, tibiae and tarsi flattened.

**Male from Cameroon.** Measurements: CL 2.20, CW 1.90, AL 3.80, AW 2.32, TL 8.20 (5.60–9.35). Length of leg segments, and total: I 2.30 + 0.75 + 1.64 + 1.91 + 1.55 = 8.15; II 2.25 + 0.86 + 1.45 + 2.16 + 1.88 = 8.60; III 1.84 + 0.90 + 0.98 + 2.23 + 2.95 = 8.90; IV 2.50 + 1.05 + 1.43 + 2.60 + 3.90 = 11.48. Carapace index 1.16; patella-tibia index 1.09.

Carapace and chelicerae brown (Fig. 5). Median ocular tubercle raised, narrow, darker in colour (Fig. 11).Chelicerae with single prolateral row of teeth, largest teeth in midsection of teeth row interspersed with smaller teeth anteriorly and posteriorly, with several denticles retrolateral of teeth row close to cheliceral base (Fig. 16). Endites elongated prolaterally, curving upwards (Fig. 11). Sternum and coxae light yellowish brown, rest of leg segments brown, fading to light yellow at tarsi. Legs weakly covered with bristles; prolateral side of patellae, tibiae and metatarsi of legs II–IV covered with spinules. Abdomen grey brown, with elongate brown scutum present in anterior half (Fig. 5). Palp with short cymbium, with rounded distal margin; embolus and conductor orientated obliquely, pointing retrolaterally and distally, not projecting beyond retrolateral cymbial margin; conductor short, broadened distally, with a prominent elongate tooth and sharp edge opposite the tooth, appearing as a second tooth; embolus short and straight (Figs 48, 49, 63–65).

#### Remarks.

Benoit’s (1967: 286, figs 1–4) drawings of a male “allotype” of *Calommata simoni* correspond with the males we have studied. However, Pocock (1903: 259) never described the male of *Calommata simoni* nor listed any males in his material studied, and thus the specimen examined by Benoit could not possibly be an allotype. The loan request to BMNH also only yielded the female lectotype of *Calommata simoni*, and no allotype or paratypes as indicated by Benoit. Benoit indeed wrongly considered the specimen used to describe the unknown sex for the first time to be the allotype, even when that occurred separately from the original description. Comments on the revalidation of *Calommata transvaalica* are provided under remarks for that species below.

Charpentier (1995) studied the biology of *Calommata simoni* in Benin, but no specimens collected by him could be traced in any collection. He collected specimens at four localities: Ayou [06°43'N, 02°07'E], Ahota [06°39'N, 02°09'E], Sè [06°28'N, 01°49'E] and Toffo [06°50'N, 02°04'E].

#### Distribution.

Widespread across tropical Africa in forests and savanna woodlands (Fig. 73).

#### Biology.

The biology of “*Calommata simoni*” was studied by Blandin (1971) and Charpentier (1995) in Côte d’Ivoire and Benin, respectively (localities listed above). However, examination of the specimens reported on by Blandin indicates that they are, in fact, *Calommata tibialis* sp. n.. In considering the habitats of the available material of *Calommata simoni* and *Calommata tibialis* sp. n., it is evident that the two species are ecologically separated, the former occurring in forests and the latter in woodland savannah. As the material collected by Charpentier (1995) could not be traced, it is impossible to determine whether he studied the biology of *Calommata simoni* or *Calommata tibialis* sp. n.. However, his indication of the habitat types at the four localities he sampled (grassland, patches of subsistence agriculture, near rivers and open ground near palm forests) suggests that the material he studied is *Calommata tibialis* sp. n. and not *Calommata simoni*. Thus, we have included biological information from their two studies under *Calommata tibialis* sp. n..

Most of the specimens studied here from the MRAC collected in Guinée, Côte d’Ivoire, Kenya, Tanzania and Congo D.R. were collected in contrasting forest types across tropical Africa, indicating that *Calommata simoni* is tolerant and adaptable to a wide range of soil, vegetation and climatic variables.

### Calommata tibialis

Fourie, Haddad & Jocqué sp. n.

urn:lsid:zoobank.org:act:E31586AD-B428-49F1-8F43-100356C90DCA

http://species-id.net/wiki/Calommata_tibialis

Figs 6, 9 17 29 31 50–52 66–68

#### Type material.

**Holotype male.** TOGO: Between Bassari and Sokodé, 09°15'N, 00°47'E, V–VII.1984, pitfalls, wooded savanna, P. Douben (MRAC 169501).

#### Paratypes.

1♂: together with holotype. CÔTE D’IVOIRE: 1♂: Kossou, 06°57'N, 04°58'W, 28.IV.1975, pitfalls, wooded savanna, R. Jocqué (MRAC 169500).

#### Other material examined.

CÔTE D’IVOIRE: 1 subadult ♀: Lamto, 06°12'N, 05°20'W, 6.III.1968, savanna with *Borassus aethiopum*, C. Girard (MRAC 232547); 1♂: Same locality, 11.II.1974, dirt road near biological station, P. Blandin (MRAC 232548).

#### Diagnosis.

The male of the species can be recognised by the carapace that is subequal in length and width (Fig. 6), the short, swollen palpal tibia, and the narrow conductor ending in a thick prominent tooth (Figs 51, 52).

#### Etymology.

The specific epithet refers to the palpal tibia of the male, which is distinctly shorter and more swollen compared to that of other African congeners.

#### Description.

**Male holotype.** Measurements: CL 2.13, CW 1.95, AL 3.45, AW 2.02, TL 6.65 (6.00–6.65). Length of leg segments, and total: I 1.98 + 0.65 + 1.35 + 1.71 + 1.63 = 7.32; II 1.80 + 0.75 + 1.10 + 1.60 + 1.80 = 7.05; III 1.49 + 0.80 + 0.75 + 1.70 + 2.45 = 7.19; IV 1.94 + 0.90 + 1.03 + 2.00 + 3.03 = 8.90. Carapace index 1.10; patella-tibia index 0.94.

**Figures 66–68.**
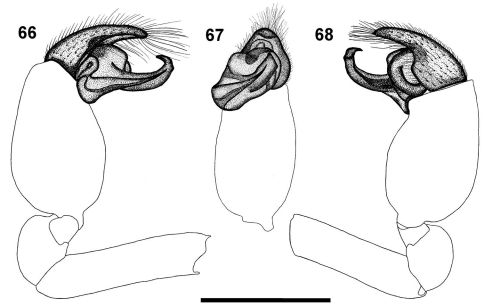
Left palp of male *Calommata tibialis* sp. n. **66** prolateral view **67** ventral view **68** retrolateral view. Scale bars: 1mm.

Carapace and chelicerae orange brown (Fig. 6). Chelicerae with single row of alternating small and medium sized teeth, gradually decreasing in size from fang base to cheliceral base, with several denticles retrolateral of teeth row near cheliceral base (Fig. 17). Eye area raised, narrow, darker in colour. Sternum and coxae yellow, remainder of legs orange, fading to pale yellow at tarsi. Legs weakly covered with bristles; prolateral side of patellae, tibiae and metatarsi of legs II–IV covered with spinules. Abdomen grey, with pale orange-brown scutum anteriorly (Fig. 6). Palp with short cymbium, with rounded distal margin; tibia shorter and broader than in the other five species, slightly longer than wide; embolus and conductor orientated obliquely, pointing retrolaterally and distally, not projecting beyond retrolateral cymbial margin; conductor narrow with a thick prominent tooth distally on its dorsal surface; embolus short and slightly curved (Figs 51, 52, 66–68).

**Female.** Unknown.

#### Remark.

The specimens may possibly have faded over time in 70% ethanol, which can only be confirmed should fresh material become available. Although a subadult female is available it will not be described as the genitalic structure cannot be studied.

#### Distribution.

Central Côte d’Ivoire and northern Togo (Fig. 73).

#### Biology.

Present data indicates that *Calommata tibialis* sp. n. occurs in woodland savannah habitats and avoids forests, where *Calommata simoni* has been collected. This may indicate some degree of ecological separation between the species but requires further study. Taking this into account we consider the studies of Blandin (1971) and Charpentier (1995) to relate to *Calommata tibialis* sp. n. and not *Calommata simoni*, as indicated by them.

Charpentier (1995) located more than 50 nests of *Calommata tibialis* sp. n. at four localities in southern Benin with quite contrasting habitat structures, including grassland, patches of subsistence agriculture, in close proximity to rivers, and open ground near palm forests. He did not indicate the occurrence of the species in forests. In one of the habitats that he found *Calommata*, the soil was described as sandy, of poor quality and relatively acidic, and covered in ‘grassland’ vegetation, similar to the habitat characteristics described by Blandin (1971) for the Lamto area, from where *Calommata tibialis* sp. n. specimens are available.

The burrow of *Calommata tibialis* sp. n. slants obliquely downwards into the soil, and was estimated to be 25–30cm deep by Blandin (1971), while Charpentier (1995) indicated a maximum depth of 21cm in a female specimen, although generally shallower in other specimens (12–19cm). The top 1–2cm of the burrow is expanded to form a chamber covered by silk webbing that is camouflaged with soil, and the spider lies in wait hanging upside-down from the web for wandering prey (Charpentier 1995). Egg sacs are suspected to hatch in May; during incubation the female spins a silk veil at the base of the chamber that is suspected to firstly allow the spider access to the chamber to capture potential prey, and secondly hide the spider and its eggs from potential parasites once they have entered the chamber (Charpentier 1995).

### Calommata transvaalica

Hewitt, 1916

species-id.net/wiki/Calommata_transvaalica

Figs 7, 8 18, 19 69–72

Calommata transvaalicus Hewitt, 1916: 180, fig. 3, pl. 26, fig. 11 **revalidated***Calommata simoni* Benoit, 1967: 283 **synonymy rejected**

#### Type material. Female holotype.

SOUTH AFRICA: *Gauteng Province*: Pretoria, Roodeplaat [25°38'S, 28°21'E], 3.IV.1915, G. van Dam (TMSA 2999 – examined).

#### Other material examined.

SOUTH AFRICA: *Gauteng Province*: 1♂: Groenkloof Nature Reserve [25°47'S, 28°12'E], 21.I.2003, reptile trap, M. Forsythe (NCA 2004/750); 1♀: Pretoria district, Hatfield [25°45'S, 28°15'E], 25.IV.1915, G. van Dam (TMSA 4639); 13♂: Zwartkoppies Farm 364, Portion 2, ca. 21km E of Pretoria, 25°45'23.6"S, 28°24'47.7"E, 1347m a.s.l., 29.X.2010, open pitfall traps, I. Engelbrecht & GDARD Field Staff (TMSA 23875). *Limpopo Province*: 1♂: Blouberg Nature Reserve, 23°00.065'S, 29°03.855'E, 29.XI.2005, searching below the knee, *Philenoptera violaceae*,A. Dawood (NCA 2009/3665); 1♀: Soutpansberg district, Blouberg, Wilhan’s Hohe [not traced], 28.VIII.1923, G. van Dam (TMSA 2772); 1♀: Same data, 29.VIII.1923 (TMSA 2773).

#### Diagnosis.

The female of this species has an additional row of two to four large prolateral teeth close to the fang base in addition to the main row of teeth, which are larger and more strongly curved than in *Calommata simoni*. The epigyne comprises a single pair of transversely oval spermathecae, while *Calommata simoni* possesses two pairs of smaller spermathecae. The male of the species shares with *Calommata meridionalis* the transversely orientated embolus and conductor, but the conductor of *Calommata transvaalica* is clearly narrower at the tip and the embolus is straight and not slightly curved as in *Calommata meridionalis*. The male chelicerae of *Calommata transvaalica* also lack the one or two large teeth found near the fang base in *Calommata meridionalis*.

#### Redescription.

**Female from Blouberg Nature Reserve.** Measurements: CL 6.72, CW 5.80, AL 13.70, AW 10.50, TL 25.20 (18.60–27.00). Length of leg segments, and total: I 1.85 + 0.60 + 0.74 + 0.85 + 0.60 = 4.64; II 1.60 + 1.05 + 0.69 + 0.90 + 0.72 = 4.96; III 1.45 + 1.28 + 0.60 + 0.80 + 0.55 = 4.68; IV 1.58 + 1.41 + 0.90 + 0.90 + 0.47 = 5.26. Carapace index 1.16; patella-tibia index 0.20.

**Figures 69–72.**
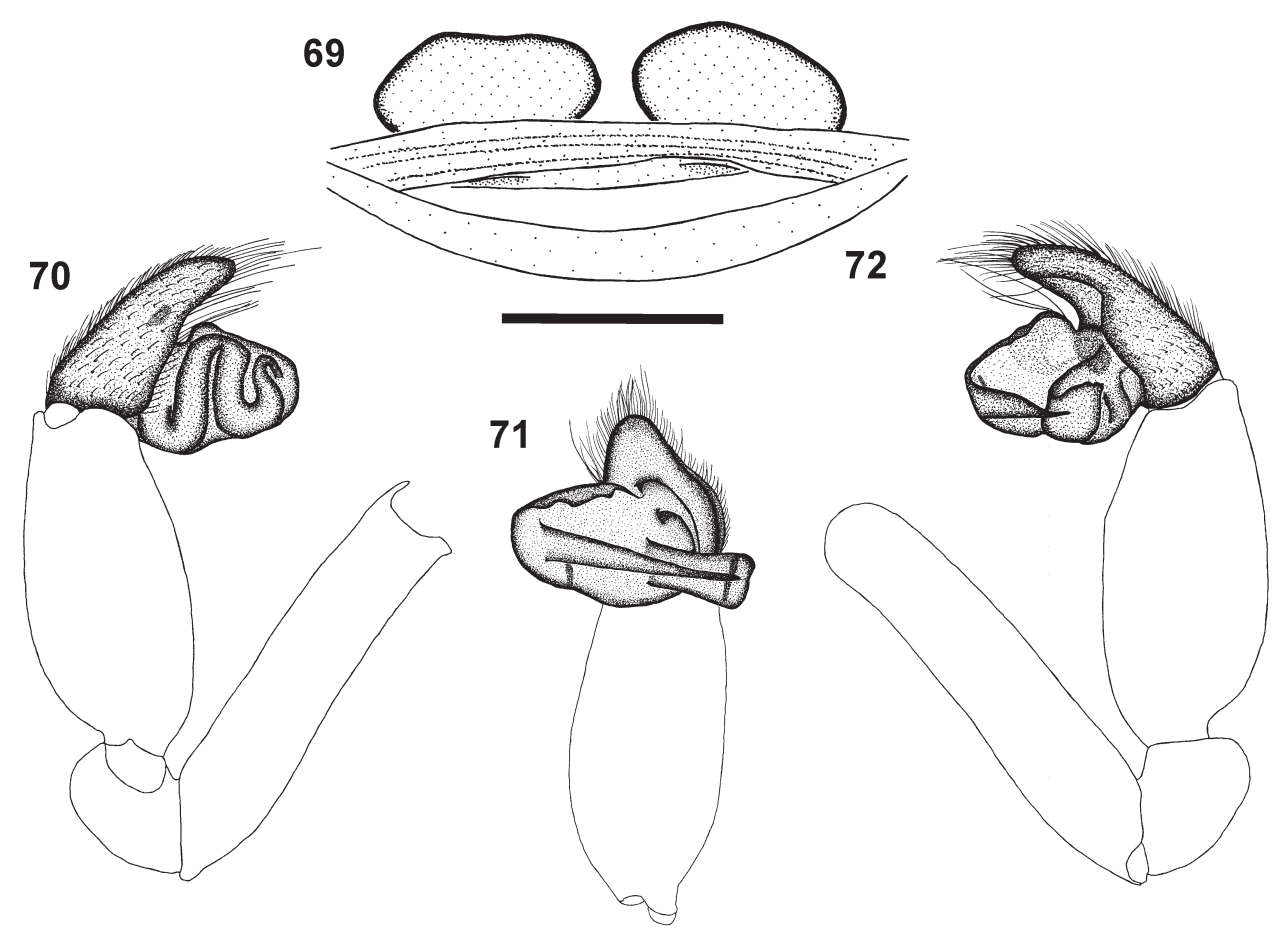
*Calommata transvaalica* Hewitt female genitalia **69** and left palp of male **70–72) 69** dorsal view **70** prolateral view **71** ventral view **72** retrolateral view. Scale bars: 1mm.

Robustly built with short legs (Fig. 7), carapace pale creamy brown. Median ocular tubercle raised, narrow, sloping sharply at fovea. Single median line running from front of median ocular tubercle to middle of chilum. Chelicerae orange, darker laterally; chelicerae with a row of two to four large teeth close to fang base, prolateral of promarginal teeth row; teeth row comprising very large teeth curved at tips, interspersed with small teeth, with extensive denticle field retrolateral of teeth row near cheliceral base (Fig. 18). Endites strongly elongated and slender prolaterally, strongly curved upwards. Sternum and legs pale yellow-brown. Legs short and stout, leg formula 4231; legs III and IV more robust than legs I and II; leg I with three to five spines on patellae and two on tibiae; leg II with few spinules on patellae and several spinules on tibiae and metatarsi; legs III and IV with spinules from patellae to tarsi (mainly dorsal and prolateral); legs II to IV covered in bristles. Abdomen globose and pale grey, with indistinct median heart marking anteriorly (Fig. 7). Epigyne forming a broad, weakly sclerotised plate ventrally, in dorsal view with single pair of large, transversely oval spermathecae (Fig. 69). Female palp short, tibiae and tarsi flattened.

**Male from Groenkloof Nature Reserve.** Measurements: CL 2.10, CW 1.85, AL 2.70, AW 1.70, TL 6.40 (6.20–6.40). Length of leg segments, and total: I 1.66 + 0.60 + 1.10 + 1.43 + 1.28 = 6.07; II 1.67 + 0.70 + 0.95 + 1.50 + 1.47 = 6.29; III 1.45 + 0.70 + 0.70 + 1.55 + 1.95 = 6.35; IV 1.85 + 0.78 + 1.05 + 1.85 + 1.90 = 7.43. Carapace index 1.14; patella-tibia index 0.81.

Carapace and chelicerae dark brown in colour (Fig. 8). Chelicerae with single row of large teeth, gradually decreasing in size from fang base to cheliceral base, without denticles near cheliceral base (Fig. 19). Carapace oval in shape. Median ocular tubercle raised, narrow, darker in colour. Sternum and coxae yellow-brown, femora, patellae and tibiae brown, metatarsi yellow-brown, tarsi yellow. Legs weakly covered with bristles; prolateral side of patellae, tibiae and metatarsi of legs II–IV covered with spinules. Abdomen dark grey, nearly black, with dark orange-brown scutum anteriorly (Fig. 8). Palp with short cymbium, cymbium tip in ventral view tapering to rounded point; embolus and conductor orientated transversely to palpal axis, pointing retrolaterally, distal ends projecting beyond retrolateral cymbial margin; conductor short, slightly broadened distally, with single sharp, curved tooth on its dorsal surface; embolus long and straight (Figs 70–72).

#### Remarks.

It is clear from the redescriptions of both sexes of *Calommata simoni*, and the redescription of the female and first description of the male of *Calommata transvaalica* provided here, that the two species have distinct differences in their somatic and genitalic morphology, most notably regarding their cheliceral dentition, number of spermathecae in the female epigyne and orientation and length of the male embolus and conductor. Consequently, we reject Benoit’s (1967) synonymy of the two species and propose the revalidation of *Calommata transvaalica*. The specimens collected by Van Dam and Roberts (1917) between Villeria and Derdepoort near Pretoria could not be traced.

The abdomen of the female holotype of *Calommata transvaalica* is damaged and therefore the specimen was not redescribed. The holotype is the smallest of the known females of this species (18.60mm long). The colour of all available female specimens has likely faded over time in 70 % ethanol.

#### Distribution.

**Figure 73.**
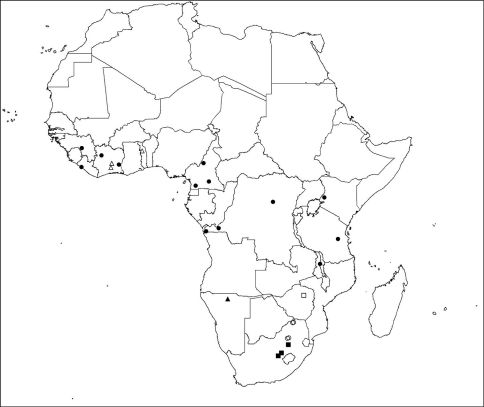
Distribution of *Calommata* species in the Afrotropical Region: *Calommata megae* (open square), *Calommata meridionalis* (solid squares). *Calommata namibica* (solid triangle), *Calommata simoni* (solid circles), *Calommata tibialis* (open triangles) and *Calommata transvaalica* (open circles).

Limpopo and Gauteng Provinces, South Africa (Fig. 73).

**Biology.** The biology of *Calommata transvaalica* was studied by Van Dam and Roberts (1917) at Roodeplaat near Pretoria, South Africa, in the days following heavy rainfall. They first detected a female (the holotype) by kicking up a tuft of grass that disclosed white webbing, which was followed downwards into the ground to locate the spider. They subsequently discovered additional nests on bare ground. They described the nests as slightly raised above the ground at the top, and then from the inner rim they were neatly rounded off, gradually sloping outwards and downwards to the level of the ground with the outer surface covered with earth that resembled the surroundings. The interior of the tube was lined with loose, highly adhesive silky webbing. They suggested that the adhesive webbing may afford the spider some protection against the intrusion of enemies. The nests were deep (22–25cm) and vertical for the greater part of their depth (Van Dam and Roberts 1917). Hewitt (1916) commented that *Calommata transvaalica* specimens had a very pronounced and objectionable odour and compared it to decomposing stable manure.

The recently collected series of males from Zwartkoppies Farm (TMSA 23875) was collected in open pitfalls without preservative from a site in open *Acacia karroo* woodland on red structured clay soils (30–45% clay in A horizon, Shortlands form). The site has a gentle slope and had recently been burned. The activity of the males appears to be related to heavy rainfall that had fallen two days prior to the collection of the males. It thus seems that males do not emerge on the night immediately following a heavy shower, but instead on the night thereafter (Ian Engelbrecht, pers. comm.).

## Discussion

*Calommata* is a small but widespread genus that is known from Africa, Israel and South East Asia. Two species are present in West Africa, one species in Central Africa and four species in southern Africa. The type of habitat seems to play a role in separating the species biogeographically. *Calommata simoni* is perhaps the most flexible in terms of habitat requirements, and occurs in forests, savannahs and grasslands in tropical Africa. In contrast, *Calommata tibialis* was only found in wooded savannahs in West Africa. In southern Africa, *Calommata megae*, *Calommata namibica* and *Calommata transvaalica* occur in the Savannah biome, but with contrasting vegetation structures and climatic variables. Lastly, *Calommata meridionalis* occurs only in the Grassland biome of central South Africa (Fig. 73).

Atypid spiders are widely regarded as being of conservation importance due to their generally specific environmental requirements, low rates of dispersal and general scarcity (Pedersen and Loeschcke 2001, Řezáč et al. 2007, Řezáč 2009). Several species are considered critically threatened or endangered and are included in various country or regional Red Data lists (e.g. Platen et al. 1996, Komposch and Steinberger 1999, Blick and Scheidler 2003, Farkač et al. 2005, Sato et al. 2007).

*Calommata transvaalica* (previously as *Calommata simoni* in South Africa) was until recently presumed nationally extinct in South Africa as the species had last been reported in the 1920’s (Dippenaar-Schoeman 2002). It was subsequently rediscovered in 2003 (Groenkloof NR) and 2005 (Blouberg NR). This species was submitted for an initial Red List assessment in April 2008, but during the process of assessment, questions arose regarding the taxonomic status of this species and it was included in the Data Deficient category for taxonomic reasons (Engelbrecht 2008). As the genus has now been revised, the South African species can be resubmitted for Red Data listing to promote conservation of these spiders. In comparing the two South African species, populations of *Calommata transvaalica* are under severe threat in the south of its range due to rapid urbanisation and habitat loss in the Gauteng Province. The recent collection of 13 males from Zwartkoppies Farm near Pretoria suggests that healthy populations do still exist in natural habitats in the province. In contrast, *Calommata meridionalis* is experiencing considerably lower threat levels (the three known localities are from undisturbed grassland) and potential agricultural expansion, especially from cultivation agriculture, perhaps represents its greatest threat. If more attention can be paid to the apparent soil preferences of South African *Calommata* in future when conducting pitfall surveys it is likely that further populations could be located in suitable habitat. As such, South African *Calommata* may serve as ideal candidates of predictive modelling due to their restricted distributions, limited knowledge of their biology, and increasing threats to their survival (see Jiménez-Valverde and Lobo 2006, Jiménez-Valverde et al. 2007).

## Supplementary Material

XML Treatment for Calommata

XML Treatment for Calommata megae

XML Treatment for Calommata meridionalis

XML Treatment for Calommata namibica

XML Treatment for Calommata simoni

XML Treatment for Calommata tibialis

XML Treatment for Calommata transvaalica
